# Supplementary material for: An integrative approach to the study of Kempnyia Klapálek, 1914 (Plecoptera: Perlidae) from Brazil: Support for the description of four new species and a basis for future studies
Source: PLoS One. 2024 Jul 17;19(7):e0305824. doi: 10.1371/journal.pone.0305824 (PMC11253944; doi:10.1371/journal.pone.0305824)
Supplement: S1 Table — Specimen vouchers with respective identification, collecting local and GenBank accession codes of COI sequences. (DOCX) [file pone.0305824.s001.docx]

**S1 Table. Voucher codes.** Specimen vouchers with respective identification, collecting local and GenBank accession codes of COI sequences

| **Species** | **Location** | **Voucher code** | **GenBank**  **access code** | **Reference**  **article** |
| --- | --- | --- | --- | --- |
| *Paragripopteryx* sp. | BR, SP, Iporanga, Parque Estadual Intervales, Rio do Carmo | MNF70 | PP704423 | This paper |
| *E. castro* | BR, MT, Nova Xavantina, Córrego da Mata | DP504 | OR496992 | Almeida *et al*. (2023) |
| *E. castro* | BR, MT, Nova Xavantina, Córrego da Mata | DP505 | OR496993 | Almeida *et al*. (2023) |
| *E. froehlichi* | BR, AM, Manaus, Igarapé do Aeroporto, Avenida do Turismo | DP754 | OR496986 | Almeida *et al*. (2023) |
| *E. froehlichi* | BR, AM, Manaus, Igarapé do Aeroporto, Avenida do Turismo | DP755 | OR496987 | Almeida *et al*. (2023) |
| *M. veneranda* | BR, SP, Salesópolis, Estação Biológica de Boracéia, Córrego Venerando | DP763 | OR496988 | Almeida *et al*. (2023) |
| *M. veneranda* | BR, SP, Salesópolis, Estação Biológica de Boracéia, Córrego Venerando | DP764 | OR496989 | Almeida *et al*. (2023) |
| *M. matogrossensis* | BR, GO, Alto Paraíso de Goiás, Cachoeira dos cristais, Ribeirão Saltador | DP589 | OR496991 | Almeida *et al*. (2023) |
| *M. matogrossensis* | BR, GO, Alto Paraíso de Goiás, Cachoeira dos cristais, Ribeirão Saltador | DP674 | OR496990 | Almeida *et al*. (2023) |
| *A. tupi* | BR, SP, Iporanga, Parque Estadual Intervales, Rio do Carmo | LP10 | MW293834.1 | Almeida and Bispo (2020) |
| *A. tupi* | BR, SP, Iporanga, Parque Estadual Intervales, Rio do Carmo | LP11 | MW293835.1 | Almeida and Bispo (2020) |
| *A. subcostalis* | BR, SP, Iporanga, Parque Estadual Intervales, Rio do Carmo | LM07 | MW293829.1 | Almeida and Bispo (2020) |
| *A. subcostalis* | BR, SP, Iporanga, Parque Estadual Intervales, Rio do Carmo | LM08 | MW293830.1 | Almeida and Bispo (2020) |
| *K. colossica* | BR, PR, Morretes, Mata Atlântica Park Hotel | LL14 | MW293839.1 | Almeida and Bispo (2020) |
| *K. colossica* | BR, PR, Morretes, Mata Atlântica Park Hotel | LL16 | MW293840.1 | Almeida and Bispo (2020) |
| *K. goiana* | BR, GO, Pirenópolis, Parque Estadual dos Pireneus, Córrego Inferno | DP555 | PP704424 | This paper |
| *K. goiana* | BR, GO, Alto Paraíso de Goiás, PARNA Chapada dos Veadeiros, Pouso Alto | DP570 | PP704425 | This paper |
| *K. gracilenta* | BR, ES, Santa Teresa, REBIO Augusto Ruschi, Córrego Bragacho | DP82 | PP704426 | This paper |
| *K. gracilenta* | BR, ES, Santa Teresa, REBIO Augusto Ruschi, Córrego Bragacho | DP83 | PP704427 | This paper |
| *K. flava* | BR, SP, São Miguel Arcanjo, Parque Estadual Carlos Botelho, Rio Bonito | LM34 | MW293845.1 | Almeida and Bispo (2020) |
| *K. flava* | BR, SP, São Miguel Arcanjo, Parque Estadual Carlos Botelho, Rio Bonito | LM35 | MW293846.1 | Almeida and Bispo (2020) |
| *K. jatim* | BR, ES, Santa Teresa, REBIO Augusto Ruschi, Córrego da Estrada | DP85 | PP704428 | This paper |
| *K. jatim* | BR, ES, Santa Teresa, REBIO Augusto Ruschi, Córrego da Estrada | DP86 | PP704429 | This paper |
| *K. mirim* | BR, MG, Poços de Caldas, Fazenda de Eucalipto | DP269 | PP704430 | This paper |
| *K. neotropica* | BR, SP, Iporanga, Parque Estadual Intervales, Rio do Carmo | LP01 | MW293848.1 | Almeida and Bispo (2020) |
| *K. neotropica* | BR, SP, Iporanga, Parque Estadual Intervales, Rio do Carmo | LP02 | MW293849.1 | Almeida and Bispo (2020) |
| *K. obtusa* | BR, SP, Salesópolis, Estação Biológica de Boracéia, Córrego Coruja | DP704 | PP704431 | This paper |
| *K. oliverai* | BR, GO, Alto Paraíso de Goiás, Cachoeira dos cristais, Ribeirão Saltador | DP585 | PP704432 | This paper |
| *K. oliverai* | BR, GO, Alto Paraíso de Goiás, Cachoeira dos cristais, Ribeirão Saltador | DP586 | PP704433 | This paper |
| *K. pirata* | BR, SP, Jundiaí, REBIO Serra do Japi, Córrego do Paraíso | DP742 | PP704434 | This paper |
| *K. pirata* | BR, SP, Jundiaí, REBIO Serra do Japi, Córrego do Paraíso | DP743 | PP704435 | This paper |
| *K. reticulata* | BR, ES, Santa Teresa, REBIO Augusto Ruschi, Córrego Entre Estradas | DP77 | PP704436 | This paper |
| *K. reticulata* | BR, ES, Santa Teresa, REBIO Augusto Ruschi, Córrego Bragacho | DP78 | PP704437 | This paper |
| *K. sazimai* | BR, MG, Jaboticatubas. PARNA Serra do Cipó, Alto Palácio, Córrego Indaia | DP693 | PP704438 | This paper |
| *K. sazimai* | BR, MG, Jaboticatubas. PARNA Serra do Cipó, Alto Palácio, Córrego Indaia | DP696 | PP704439 | This paper |
| *K. tamoya* | BR, SP, Campos do Jordão, Parque Estadual de Campos do Jordão, Córrego Galharada | DP314 | PP704440 | This paper |
| *K. tenebrosa* | BR, SC, Blumenau, PARNA Serra do Itajaí, Parque das Nascentes, Trilha do Morro do Sapo, Córrego da Placa | DP341 | PP704441 | This paper |
| *K. tijucana* | BR, RJ, Mangaratiba, Ilha da Marambaia, Rio Marambaia | DP232 | PP704442 | This paper |
| *K. tijucana* | BR, RJ, Mangaratiba, Ilha da Marambaia, Rio Marambaia | DP233 | PP704443 | This paper |
| *K. tupinamba* | BR, SP, Campos do Jordão, Parque Estadual de Campos do Jordão, Córrego Galharada | DP307 | PP704444 | This paper |
| *K. tupinamba* | BR, SP, Campos do Jordão, Parque Estadual de Campos do Jordão, Córrego Galharada | DP309 | PP704445 | This paper |
| *K. umbrina* | BR, SP, Salesópolis, Estação Biológica de Boracéia, Córrego Venerando | DP761 | PP704446 | This paper |
| *K. umbrina* | BR, SP, Salesópolis, Estação Biológica de Boracéia, Córrego Coruja | DP823 | PP704447 | This paper |
| ***K. guarany* sp. nov.** | BR, SC, Blumenau, PARNA Serra do Itajaí, Parque das Nascentes, Encontro das águas | DP351 | PP704448 | This paper |
| ***K. guarany* sp. nov.** | BR, SC, Blumenau, PARNA Serra do Itajaí, Parque das Nascentes, Encontro das águas | DP349 | PP704449 | This paper |
| ***K. tupiniquim* sp. nov.** | BR, ES, Santa Teresa, REBIO Augusto Ruschi, Córrego da Estrada | DP71 | PP704450 | This paper |
| ***K. una* sp. nov.** | BR, SP, Iporanga, Parque Estadual Intervales, Rio do Carmo | PPI 5 | MW293844.1 | Almeida and Bispo (2020) |
| ***K. zwicki* sp. nov.** | BR, ES, Santa Teresa, REBIO Augusto Ruschi, Córrego Bragacho | DP56 | PP704451 | This paper |
