# Supplementary material for: An integrative approach to the study of Kempnyia Klapálek, 1914 (Plecoptera: Perlidae) from Brazil: Support for the description of four new species and a basis for future studies
Source: PLoS One. 2024 Jul 17;19(7):e0305824. doi: 10.1371/journal.pone.0305824 (PMC11253944; doi:10.1371/journal.pone.0305824)
Supplement: Attachment — Submitted filename: Response to Reviewers.docx [file pone.0305824.s004.docx]

Rebuttal Letter

Dear Editor

We would like to submit the revised manuscript “An integrative approach to the study of *Kempnyia* Klapálek, 1914 (Plecoptera: Perlidae) from Brazil: Support for the description of four new species and a basis for future studies” to continue the publication process in “Plos One”.

We would like to thank the Editor and the two reviewers for their suggestions. We considered all the suggestions given by the reviewers and all the requirements questioned by the Editor. We would just like to highlight some important points:

1. About the presence of financing in the acknowledgments: This has been corrected and the information on financing obtained is correctly entered into the system.

2. 2. About the maps: The shapefile used was obtained from SimpleMappr, an online and free-to-use platform. In any case, we added SimpleMappr to the Materials and Methods and added to this Rebuttal Letter the excerpt that qualifies SimpleMappr as a free platform, which is present on the platform creator's website (https://www.simplemappr.net/#tabs=6).

“All versions of SimpleMappr map data found on this website are in the Public Domain. You may use the maps in any manner, including modifying the content and design, electronic dissemination, and offset printing. The primary author, David P. Shorthouse has waived all copyright, related or neighboring rights, and financial claim to the maps and invites you to use them for personal, educational, and commercial purposes. No permission is needed to use SimpleMappr.”

Best regards

Authors
