# Supplementary material for: An integrative approach to the study of Kempnyia Klapálek, 1914 (Plecoptera: Perlidae) from Brazil: Support for the description of four new species and a basis for future studies
Source: PLoS One. 2024 Jul 17;19(7):e0305824. doi: 10.1371/journal.pone.0305824 (PMC11253944; doi:10.1371/journal.pone.0305824)
Supplement: Attachment — Submitted filename: Response to Reviewers.docx [file pone.0305824.s006.docx]

Rebuttal Letter

Dear Editor

We would like to submit the revised manuscript “An integrative approach to the study of *Kempnyia* Klapálek, 1914 (Plecoptera: Perlidae) from Brazil: Support for the description of four new species and a basis for future studies” to continue the publication process in “Plos One”.

We would like to thank the Editor and the two reviewers for their suggestions. We considered all the suggestions given by the Reviewers and Editor.

Best regards

Authors
